# 

**Published:** 1888-04-28

**Authors:** 


### CONTENTS.

**The Elberfeld System** - 53

**Hospital Saturday Movement** - 54

**The Doctor's Pulpit.**—Constitution Building - 55

**Aids to Nursing.** By M. M. Basil, M.A., M.B. - 56

**Home Colonisation** - 57

**The Editor's Letter-Box.**—A Hard Case - 58

**A Plea for the London Hospital.** By the Rev. J. F. Kitto, M.A., Rector of St. Martin-in-the-Fields - 59

**Notes and News** - 60

**Everybody's Page** - 62

**Annotations.**—A Homœopathic Earl —Something like a Secretary.—Hospital Patients and Stimulants - 63

**Turning over New Leaves.**—"Our Hundred Days in Europe"—"Human Anatomy and Physiology" - 64

**An Ishmaelite.** By Leslie Keith, Author of "The Chilcotes," "East and West," etc. - 65

**London Charities:** Quinn's and Graham's Bequests - 67

**The Student's Column** - 68

**Amusements with Profit for all Ages.**—For Men and Women.—Children's Corner.—Puzzles, etc. - 68

**EXTRA SUPPLEMENT.—THE NURSING MIRROR.**

En Passant—The Pay of Private Nurses.—*The Lancet.*—Examinations.—Nursing in Canada.—Twenty per Cent.—Hospital Nurses in India - ix
The National Pension Fund - x
A District Matron's Diary - xi
Anecdotes of Personal Adventure - xi
Everybody's Opinion.—A Case for the Pension Fund.—The British Nurses' Association - xii
Notes and Queries - xii
Appointments—Vacancies.—Exchange and Mart - xii

